# Non-canonical IL-2 signaling facilitates peripheral regulatory T cell differentiation

**DOI:** 10.64898/2026.10.07.756301

**Published:** 2026-10-14

**Authors:** Amelia Meecham, Sarah Hyun Ji Kim, Mikhael Haruo Fernandes de Lima, Michelle Tang, Bryan Liao, Samuel Chan, Ho-sup Lee, Li-Fan Lu, Ziyue Zhang, Caroline Jefferies, Monica Guma, Miguel-Alejandro Lopez-Ramirez, Mark H. Ginsberg

## Abstract

Non-canonical IL-2 signaling, termed **M** onoclonal antibody or **M** atrix-directed **A** lternative **C** ytokine **S** ignaling (MACS), is mediated by a CD25–chemokine receptor complex and operates in concert with the canonical JAK-STAT5 pathway; however, its role in T cell homeostasis remains poorly defined. Here, we uncover a role for MACS in regulatory T cell (Treg) differentiation and functional specification. We generated *Il2* ^E66K */E66K*^ mice in which CD25–CCR7 assembly is disrupted and IL-2-induced STAT5 phosphorylation is preserved. Immunophenotyping analysis by flow cytometry revealed that MACS deficiency reduces the accumulation of Nrp1 ^-^ Foxp3 ^+^ Tregs in gut-associated lymphoid tissues from 24-weeks-old *Il2* ^E66K/E66K^ mutant mice. Single-cell transcriptomics, functional suppression assays, and *in vitro* differentiation revealed that MACS engagement drives Treg differentiation and sustains effector Treg programs—marked by robust IL-10 expression—in part via an AP-1-dependent transcriptional network. These findings establish IL-2 MACS as a distinct signal directing peripheral immune tolerance and highlight a potential node for targeted immunomodulation.

**One Sentence Summary:**

Non-canonical IL-2 signaling facilitates gut regulatory T cell development and suppressive function.

## Full Text Availability

The license terms selected by the author(s) for this preprint version do not permit archiving in PMC. The full text is available from the preprint server.

